# Effects of Eleutherococcus Extract Mixture on Endochondral Bone Formation in Rats

**DOI:** 10.3390/ijms20051253

**Published:** 2019-03-12

**Authors:** Donghun Lee, Sung Hyun Lee, Namhoon Cho, Young-Sik Kim, Jungbin Song, Hocheol Kim

**Affiliations:** 1 Department of Herbal Pharmacology, College of Korean Medicine, Gachon University, 1342 Seongnamdae-ro, Sujeong-gu, Seongnam-si, Gyeonggi-do 13120, Korea; dlee@gachon.ac.kr; 2 Korea Institute of Science and Technology for Eastern Medicine (KISTEM) NeuMed Inc., 88 Imun-ro, Dongdaemun-gu, Seoul 02440, Korea; lsh@neumed.co.kr; 3 Department of Herbal Pharmacology, College of Korean Medicine, Kyung Hee University, 26 Kyungheedae-ro, Dongdaemun-gu, Seoul 02447, Korea; gmpops21-kj@daum.net (N.C.); yjbsik@gmail.com (Y.-S.K.)

**Keywords:** *Eleutherococcus sessiliflorus*, germinated barley, endochondral bone formation, growth plate, IGF1

## Abstract

Eleutherococcus extract mixture (EEM) is an herbal mixture of dried stem of *Eleutherococcus sessiliflorus* and germinated barley, which has been highly effective, in previous screening and among the traditional medicines to tonify innate *qi* and acquired *qi*, respectively. In this study, we investigate the effects of EEM on endochondral bone formation. Female adolescent rats were given EEM, growth hormone or vehicle for 10 days. Tetracycline was intraperitoneally injected to light the fluorescent band 72 h before sacrifice to determine endochondral bone formation. In order to evaluate endocrine or paracrine/autocrine mechanisms, expressions of insulin-like growth factor 1 (IGF1), insulin-like growth factor binding protein 3 (IGFBP3), or bone morphogenetic protein 2 (BMP2) were evaluated after EEM administration in liver or growth plate (GP). EEM oral administration significantly increased endochondral bone formation and proliferative and hypertrophic zonal heights of tibial GP. EEM also upregulated hepatic IGF1 and IGFBP3 mRNA expressions, and IGF1 and BMP2 expressions in GP. Taken together, EEM increases endochondral bone formation through stimulating proliferation and hypertrophy with upregulation of hepatic IGF1 and IGFBP3 expressions. Considering immunohistochemical studies, the effect of EEM may be due to increased local IGF1 and BMP2 expression in GP, which may be considered growth hormone (GH)-dependent endocrine and autocrine/paracrine pathways.

## 1. Introduction

Short stature is defined as the height of individuals who are shorter than two standard deviations compared with the mean height of age, gender, and population [1]. Short children may be at risk of psychosocial issues such as poor self-esteem, lower social competence, or being victims of bullying [2,3,4,5,6]. Relative short people, although they do not belong to the definition of short stature, are reported to have a poor health-related quality of life [7]. Relative short adults usually have thinner blood vessels and are more likely than taller individuals to develop coronary artery disease [8,9].

Only one-fifth of individuals are short stature due to certain diseases, including growth hormone (GH) deficiency, while the remaining four-fifths are short stature with enough GH, called idiopathic short stature (ISS) [1]. GH therapy has long been accepted as a method of treating the pathological condition of short stature, but GH treatment in ISS is controversial [10,11]. In the case of ISS, the height increase in children treated with 4–7 years of long-term GH was just 3.5–7.5 cm and the estimated cost of GH treatment for adult height increase is approximately 9000–60,000 dollars/cm [12,13]. Another concern regarding GH therapy is that it could have adverse psychosocial consequences due to the treatment burden resulting from daily subcutaneous injections [14]. Therefore, it is necessary to develop a growth promoting substance that can be administered orally without injection and that is less expensive.

According to traditional Korean medicinal theory, the process of height growth in children is influenced by *qi*. *Qi* is regarded as the root of life whether it is inherited from the parents (innate *qi*) or acquired from the essence such as food or air (acquired *qi*). Therefore, we selected medicinal herbs from the Dongeuibogam that have been used to tonify innate *qi* or acquired *qi*. We screened traditional medicinal herbs to discover the safe oral growth stimulant using an established animal model which could assess the endochondral bone formation using tetracycline in rats [15,16,17]. Eleutherococcus extract mixture (EEM) is an herbal mixture of dried stem of *Eleutherococcus sessiliflorus* and germinated barley, which was has been highly effective in previous screening, and among the traditional medicines to tonify innate *qi* and acquired qi, respectively.

*E. sessiliflorus*, which belongs to the Araliaceae family, is found extensively in the Far East of Russia and Northeast Asian countries such as Korea, Japan, and China. The roots and stems are traditionally used to tonify innate *qi* by strengthening the muscles and bones. *E. sessiliflorus* has been used in oriental medicine for the treatment of rheumatoid arthritis, inflammation, and diabetes [18,19,20]. Chiisanoside is one of the main compounds of the Eleutherococcus species and is reported to have anti-inflammatory, antihepatotoxic, antidiabetic, and antiviral activities [21,22]. The Eleutherococcus species are reported to have bone remodeling and bone formation effects on in vitro and in vivo models [23,24].

Germinated barley, the germinated seed of *Hordeum vulgare* L., is widely used as a raw material for making beer and beverage, and at the same time, it is used as a medicinal herb in Korean medicine. As a medicinal herb it has been used to tonify acquired *qi* by treating loss of appetite and diarrhea because of its traditional oriental medical effects such as promoting digestion and invigorating the spleen. Germinated barley contains hordenine, p-coumaric acid, ferulic acid, and β-glucan known to be responsible for various biological activities. Previous studies have reported on the use of germinated barley for enhancing digestion, reducing blood glucose, treating hyperprolactinemia, inhibiting melanogenesis, and treating precocious puberty [25,26,27,28,29,30]. Especially, Sawadogo et al. also reported that aqueous extracts of the residue from germinated barley after brewing induced growth hormone secretion in ewes and cows [31].

To observe the effects of EEM on the rate of endochondral bone formation, we used tetracycline, as an intravital marker, staining the afresh formed bones in the growth plate (GP) [32]. We also analyzed the effects of EEM on zonal heights of the proximal tibial GP. In order to evaluate the possible endocrine or paracrine/autocrine mechanism by which EEM can exert its growth-stimulating effect, we assessed insulin-like growth factor-1 (IGF1) and bone morphogenetic protein 2 (BMP2) expressions in GP, and also the IGF1 and insulin-like growth factor binding protein 3 (IGFBP3) mRNA expressions in liver.

## 2. Results

### 2.1. HPLC Analysis of EEM Extract

**Figure 1:**
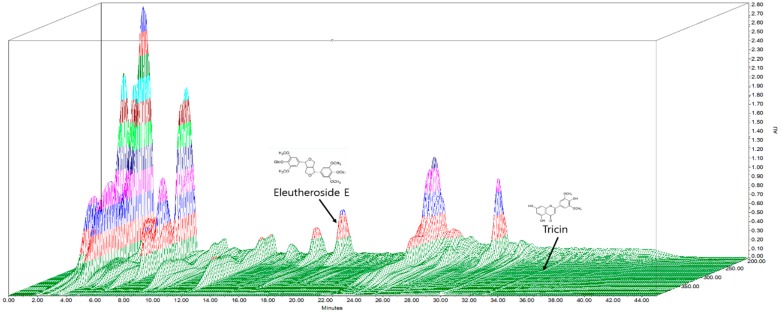
The three-dimensional high-performance chromatography of eleutherococcus extract mixture (EEM).

EEM extract was standardized to contain more than 0.005% tricin and 0.153% eleutheroside E, respectively. Figure 1 shows a representative three-dimensional chromatogram of the batch used for this study which contained 0.006% tricin and 0.170% eleutheroside E.

### 2.2. Effects on Endochondral Bone Formation

**Figure 2:**
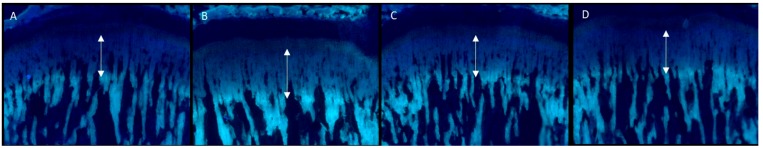
Representing fluorescent microscopic pictures of the proximal tibial growth plate (GP). The double arrows indicate the distances between the chondrocyte-bone junction and the proximal endline of the tetracycline label which represent bone growth during 72 h. (**A**) control group, (**B**) rhGH 200 μg/kg group, (**C**) EEM 50 mg/kg group, and (**D**) EEM 200 mg/kg group. Scale bar is 200 μm.

**Figure 3:**
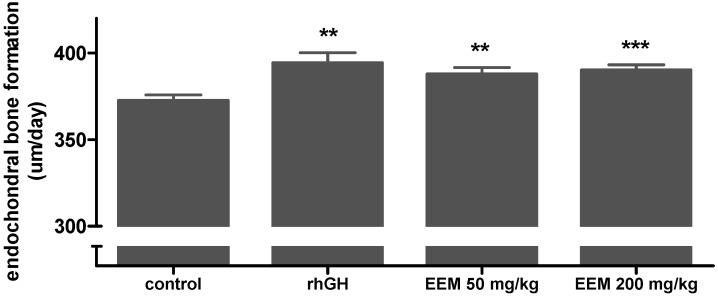
Effects of EEM on endochondral bone formation in Growth Plate (GP) of proximal tibial. Each value is the mean ± SEM. The number of rats is 53–55 per group; ** *p* < 0.01 and *** *p* < 0.001 vs. control by one-way ANOVA, Dunnett’s test.

To evaluate the effects of EEM on endochondral bone formation, tetracycline fluorescent labeling was used to bind the afresh formed bones on the GP. The length of the double arrows indicates the amount of bone growth in GP for 72 h (Figure 2). Compared to the control group, the length was increased by oral administration of EEM at a dose of 200 mg/kg. Figure 3 shows the rate of endochondral bone formation of each group. Endochondral bone formation of the control group was 372.8 ± 3.0 μm/day, and of the rhGH group was 394.4 ± 5.8 μm/day. EEM 50 mg/kg and 200 mg/kg significantly increased the endochondral bone formation, which were 388.0 ± 3.6 μm/day and 390.3 ± 2.9 μm/day, respectively, as compared to the control group.

### 2.3. Effects on GP Height

**Figure 4:**
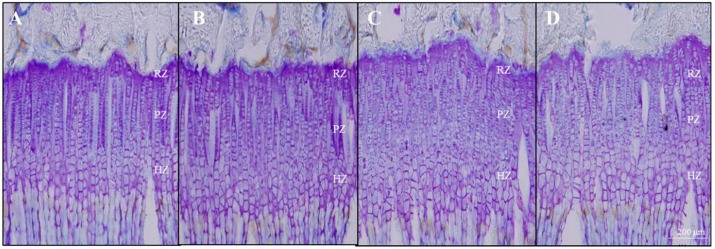
Representing microscopic pictures of Cresyl Violet stained chondrocytes of the proximal tibial GP in rats. (**A**) control group, (**B**) rhGH 200 μg/kg group, (**C**) EEM 50 mg/kg group, and (**D**) EEM 200 mg/kg group. Scale bar is 200 μm.

GP height of the proximal tibia was measured after CV staining. The microscopic pictures of sections stained with CV are shown in Figure 4. The mean overall GP height of the control group was 350.3 ± 19.6 μm and that of the rhGH group was 365.4 ± 17.3 μm. Oral administration of 50 mg/kg and 200 mg/kg of EEM significantly increased the GP height exhibiting 368.2 ± 27.7 μm and 368.0 ± 31.4 μm respectively, as compared to the control group. Particularly, proliferative zone (PZ) and hypertrophic zone (HZ) heights were significantly increased in EEM groups, respectively (Table 1).

### 2.4. Effects on IGF1 and BMP2 Expressions in GP

**Figure 5:**
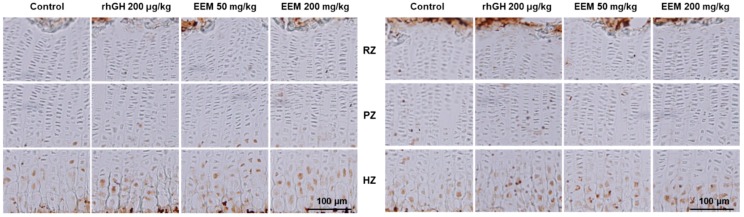
Immunohistochemical localizations of IGF1 and BMP2 on the proximal tibial GP in rats. RZ: Resting zones, PZ: Proliferative zones, HZ: Hypertrophic zones. Scale bar is 100 μm.

Protein expressions of IGF1 and BMP2 were evaluated with antigen-specific immunohistochemical staining in the proximal tibial GP. Administration of EEM or rhGH markedly increased the intensity of IGF1 expressions in PZ and HZ compared to the control. BMP2 expressions were also remarkably higher in EEM or the rhGH groups (Figure 5).

### 2.5. Effects on IGF1 and IGFBP3 mRNA Expressions in Liver 

**Figure 6:**
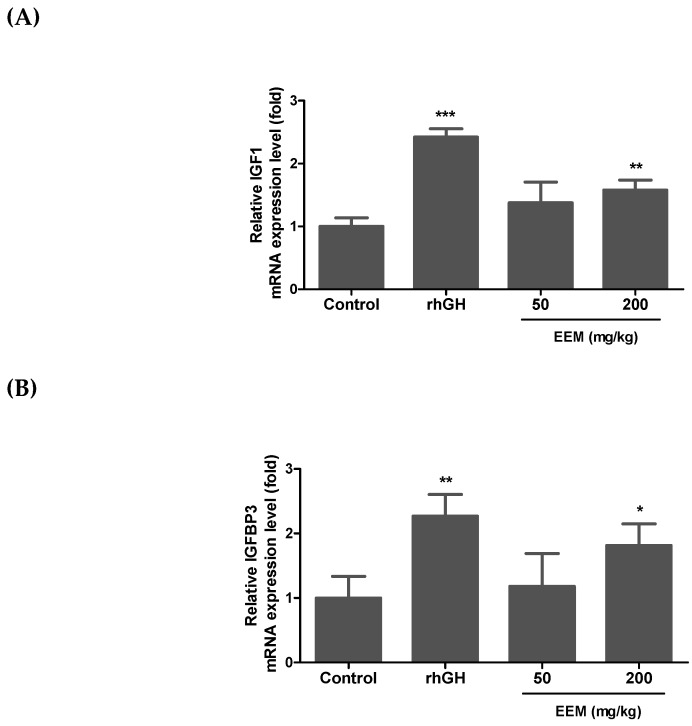
Relative expressions of IGF1 and IGFBP3 mRNA in liver. The relative transcription levels of IGF1 (**A**) and IGFBP3 (**B**) were analyzed by applying to GAPDH internal control; * *p* < 0.05, ** *p* < 0.01, *** *p* < 0.001.

Serum IGF1 is mainly synthesized in the liver by the stimulation of GH [33]. Thus, liver IGF1 and IGFBP3 mRNA expressions were measured by quantitative real-time PCR to assess whether EEM can affect circulating IGF1. Although the increase was less than rhGH, EEM 200 mg/kg significantly increased liver IGF1 and IGFBP3 mRNA expressions at 1.6 and 1.8 times, respectively, as compared to the control (Figure 6).

## 3. Discussion

In adolescent female rats, 30% EtOH extracts of EEM for 10 days significantly increased endochondral bone formation and GP height of proximal tibia, as compared to the control group. EEM also increased the IGF1 and BMP2 expressions in the HZ of the GP, and the IGF1 and IGFBP3 mRNA expressions in liver.

EEM 50 mg/kg and 200 mg/kg increased the endochondral bone formation of tibia by 4.1% and 4.7%, respectively, as compared to the control group. Endochondral bone formation rates of the control and the rhGH groups were 372.8 µm/day and 394.4 µm/day, respectively, and the rhGH group increased endochondral bone formation of tibia by 5.8% as compared to control group, previous studies found similar results using the same experimental model [15,17]. Tetracycline fluorescent labeling of bone was used to assess the endochondral bone formation of tibia. When tetracycline is administered intraperitoneally, it is deposited on the newly calcified bones, binds to calcium molecules, and causes a fluorescent label [34]. The distance between the cartilage-bone junction and the fluorescent line refers to the rate of endochondral bone formation, which means the increased bone length in the growth plate within a fixed unit of time [32]. The long bones of the human leg compose almost half of the adult height and endochondral bone formation of long bone is commonly used for assessing height growth processes [35,36]. The tibia is one of the long bones that has the highest correlation with human height, so it is known as the most representative bone for predicting height [36]. The result suggests that EEM increases endochondral bone formation, which is important for the height growth process.

EEM 50 mg/kg and 200 mg/kg significantly increased the length of GP by 5.1% as compared to the control group, especially that of PZ and HZ of the GP. GP height is known to be one of the main indices reflecting the endochondral bone formation because endochondral bone formation is determined by the complex interaction occurring in the GP [37]. GP can be classified histologically into three zones: resting zone (RZ), PZ, and HZ [38,39]. Since the RZ is quite small, most of the GP height is composed of the PZ and the HZ in both rats and humans [40,41]. Chondrocytes undergo clonal expansion in PZ and the rapid mitosis rate is an important factor in determining endochondral bone formation which results in proliferative zone height [42]. Hypertrophy of chondrocytes is also an essential step between proliferation and ossification [43]. It has been reported that there is a high correlation between endochondral bone formation and height of hypertrophic zone, regardless of the type of long bones or animal species [43,44,45]. The results suggest that EEM increases the height of GP, which can be interpreted as increasing proliferation and hypertrophy of GP chondrocytes.

EEM 50 mg/kg and 200 mg/kg increased local IGF1 and BMP2 expressions in the hypertrophic zone of tibial GP. Bone growth occurs through proliferation and hypertrophy of the GP due to locally produced IGF1 stimulated directly by GH, or liver-derived circulating IGF1 stimulated by GH [46,47]. The local IGF1, mainly produced by serum GH, binds to the IGF1 receptor of the cell membrane in the GP, similar to circulating IGF1 [48]. The local IGF1 plays a role in mediating the endochondral bone formation by direct action of GH [49,50]. In association with other factors affecting GH, BMP2 stimulates bone formation through inhibiting noggins, which suppresses bone tissue generation, and also promotes bone growth by increasing chondrocyte proliferation and hypertrophy via the regulation of the retinoid pathway [51,52,53]. Recently, systemic GH has been reported to induce BMP2 mRNA expression in the GP while systemic IGF1 does not change its mRNA expression [54]. These results indicate that the increment of BMP2 production in the GP might be the cause of the stimulating effect of GH. All these results indicate that the growth stimulatory effect of EEM appears to be due to local IGF1 and BMP2 production in GP activated by GH.

EEM 50 mg/kg and 200 mg/kg increased hepatic IGF1 and IGFBP3 mRNA expression 1.6 and 1.8 times compared to those of the control, respectively. Liver is the primary source to produce circulatory IGF1 and IGFBP3 in response to GH [55]. In mammals, about four-fifths of circulating IGF1 is bound with IGFBP3 to increase the half-life of IGF1 and to deliver IGF1 to its receptor [56]. A decline of IGFBP3 weakens its IGF1 protective ability. For these reasons, serum levels of IGF1 and IGFBP3 are considered to be excellent biochemical parameters that reflect the time-varying levels of [57,58]. The positive effect of EEM on the expression of IGF1 or IGFBP3 is consistent with previous studies that report the stimulating effects of eleutheroside B, E of *E. sessiliflorus*, and β-glucan and arginine of germinated barley on plasma IGF1 or IGFBP3 level [42,59,60,61,62,63,64]. Collectively, these findings suggest that the effects of EEM on endochondral bone formation might be stimulated by circulatory IGF1 and IGFBP3 by the stimulation of GH.

Our study has a limitation in that it did not measure osteoclast activity. Osteoclast activity affects the growth plate height because it regulates the mineralization of hypertrophic chondrocytes [47]. *Eleutherococcus senticosus*, which contains active ingredients similar to *E. sessiliflorus*, has been reported to decrease the receptor activator of nuclear factor-κB ligand (RANKL)induced osteoclast differentiation and bone resorption [65,66]. Germinated barley has also been shown to inhibit RANKL-induced osteoclast formation and bone-resorbing activity in the early-to-late stages of osteoclastogenesis [67]. On the basis of these previous studies, it is assumed that inhibition of osteoclast activity might partially contribute to the EEM-mediated enlargement of the growth plate. Further studies are needed to elucidate the effects of EEM on osteoclast activity.

Our group have continuously screened herbs to evaluate the growth-stimulating effects within the vivo model. Compared with our previously published studies, in this study we adopted advanced experimental design as follows. Rats of identical age (32 days old) were used because the bone growth rate of rats varies excessively depending on their age [68]. In addition, female rats were chosen, because compared to male rats, they have a more-steady and continuous GH secretion. [69,70]. To improve accuracy, the effects of EEM on endochondral bone formation was measured for 72 h after a 10-day administration, unlike previous experiments in which the effects were measured for 48 h after a four day administration [15,16,17]. In addition, sagittal cutting was used instead of coronal cutting to reduce variations of bone sections. Unlike animal models for disease research, this model has had to continually improve its design because it has to identify the effects in normal rats. Unlike previous studies which did not perform dose-dependent studies [16] or measure the expression of growth factors in the liver [15,17], in this study EEM showed a significant effect on these parameters. EEM is the promising candidate of our group derived from data using more than 50 rats per group to confirm the effects on endochondral bone formation, while previous studies confirmed the effects using 8 to 14 rats per group [15,16,17]. It is also meaningful that the results revealed the effects of using herbs which are widely consumed as food. Interestingly, known active compounds of EEM are not similar with those of substances that we have previously shown to stimulate endochondral bone formation, chondrocyte proliferation, or serum growth factors [15,16,17]. Many studies have suggested that synergistic effects can be achieved by combining different herbal compounds [71,72]. Further research is needed to reveal possible synergy between different herbal extracts.

In conclusion, EEM stimulates endochondral bone formation promoting proliferation and hypertrophy of chondrocytes by the upregulation of hepatic IGF1 and IGFBP3 expressions. Considering the immunohistochemical studies, the effects of EEM may also be due to the increment of IGF1 and BMP2 expressions in local GP, which may be regarded as a function of GH-dependent endocrine and auto/paracrine pathways. Taken together, the results provide reasonable evidence that EEM would be a promising candidate for short stature children.

## 4. Materials and Methods

### 4.1. Plant Material

Dried stem bark of *E. sessiliflorus* was purchased from Hanjungmuyuk (Xian, China) and germinated seed of *H. vulgare* was purchased from Kimpomaegasigpum (Seoul, Korea). They were confirmed by Professor Hocheol Kim, Kyung Hee University College of Korean medicine, where the voucher specimens (#HP562 and #HP561) were deposited on 18 May 2016 and 12 May 2016, respectively.

### 4.2. Sample Preparation and Quantitative Analysis

*E. sessiliflorus* and *H. vulgare* were pulverized at the same weight ratio of 10 g each, and then extracted with 8 times the sample weight of 30% EtOH under reflux at 80–90 °C for 5 h. After 5 h of extraction, add 3 times of 30% EtOH under reflux at 80–90 °C for 2 h. The filtered and concentrated extract was lyophilized. The yield was 5.5%. The quantitative analysis of EEM was carried out by a HPLC Alliance system, consisting of a 2695 pump and a 2998 photodiode array detector (Waters, Milford, MA, USA). Separation was done at 40 °C on C_18_ column (Waters, Milford, MA, USA; 250 × 4.6 mm i.d., 5 μm). Mobile phase composition was 0.1% phosphoric acid in distilled water (A) and acetonitrile (B) with a flow rate of 1.0 mL/min. Gradient elution was as follows: 0–18 min, 12% solvent B; 18–20 min, 17% solvent B; 20–35 min, 32% solvent B; 35–36 min, 12% solvent B; and 35–45 min, 12% solvent B.

### 4.3. Animals

Female adolescent Sprague–Dawley rats (25 days old) were supplied by Samtako (Korea). Experimental procedures were carried out in accordance with the guidelines of Kyung Hee University Animal Care and Use Committee (KHUASP[SE]-13-028), approved on 5 November 2013. The animal room was maintained at a temperature of 22 ± 1 °C, and a light of 12 h. Every animal was given food and water ad libitum.

### 4.4. Treatment

Rats were randomly divided into 4 groups and adapted for 7 days before the treatment. The four groups were the control, EEM 50 mg/kg, EEM 200 mg/kg and recombinant human GH (rhGH) 200 μg/kg. Vehicle, EEM 50 mg/kg or EEM 200 mg/kg were given twice daily (08:30 and 20:30) by oral gavage and rhGH 200 μg/kg (Eutropin, LG, Seoul, Korea) was given once daily by (08:30) subcutaneous injection for 10 successive days. Rats were sacrificed to analyze on day 11.

### 4.5. Endochondral Bone Formation

Rats received intraperitoneal injections of tetracycline hydrochloride 72 h before sacrifice (20 mg/kg, Sigma, St. Louis, MO, USA). The dissected tibias were fixed with 4% para-formaldehyde, and then decalcificated in 50 mM ethylene diaminetetra acetic acid solution (Sigma). After dehydration in 30% sucrose, the dehydrated bones of the proximal part were sagittally sectioned at a thickness of 40 μm with a cryostat (CM3050 S, Leica, Nussloch, Germany). The daily rate of endochondral bone formation was calculated by measuring the length between the epiphyseal end line of the GP and the proximal endpoint of the fluorescence line, and dividing the distance by 3. Fluorescence line was observed by a fluorescence microscope (BX50, Olympus, Tokyo, Japan) and the distance was calculated with Image J (NIH, Bethesda, MD, USA). Three different researchers completed assessments to prevent the possibility of individual differences.

### 4.6. GP Height

The GP height was measured using Cresyl violet (CV, Sigma) staining of chondrocytes and Image J. The height of the entire GP and that of RZ, (PZ and HZ was measured at 3 different points. The PZ was measured from flat-chondrocytes along with the long axis of the bones. The HZ was measured from chondrocytes with expanded nucleus and cytoplasm that were easily differentiated by size. The RZ was calculated from the overall height of GP minus PZ and HZ.

### 4.7. Immunohistochemistry

After pretreatment as previously described [15], the sagittal sections were incubated overnight with rabbit IGF1 and goat BMP2 primary-antibody (1/200, Santa Cruz, Dallas, TX, USA), respectively. After washing, the sections were incubated for an hour with rabbit secondary-antibody (1/200, Jackson, West Grove, PA, USA). After washing, sections were incubated with avidin-biotin complex reagent (1/100, Vector, USA) for 1 h. Sections were stained with a 0.05% 3,3-diaminobenzidine (Sigma) with hydrogen peroxide.

### 4.8. Real-Time Quantitative Polymerase Chain Reaction (PCR)

After sacrifice, the liver was immediately removed, rinsed, and stored at −80°C. Total RNA was extracted from hepatocytes by QIAzol (Qiagen, USA) and transcribed into cDNA using the transcription kit (AppliedBiosystems, USA). Quantitative PCR was performed with a real-time PCR system (AppliedBiosystems, USA) followed by 40 cycles of 95 °C for 10 min, 95 °C for 15 s, and 60 °C for 60 s. Primer was designed in Bioneer (Korea): *IGF1* (GenBank M15481), forward 5′-GCTATGGCTCCAGCATTCG-3′ and reverse 5′-TCCGGAAGCAACACTCATCC-3′; *IGFBP3* (GenBank NM_012588), forward 5′-GGAAAGACGACGTGCATTG-3′ and reverse 5′-GCGTATTTGAGCTCCACGTT-3′; and glyceraldehyde−3−phosphate dehydrogenase (*GAPDH*, GenBank NM_017008), forward 5′-TGGCCTCCAAGGAGTAAGAAAC-3′ and reverse 5′-CAGCAACTGAGGGCCTCTCT-3′. Conditions were repeated 40 times at 95 °C for 10 m, 95 °C for 15 s, 60 °C for 60 s. Relative quantification was performed by normalizing using *GAPDH* expression using the delta-delta Ct method.

### 4.9. Statistical Analysis

The effect was analyzed using GraphPad Prism 5 (GraphPadSoftware, San Diego, CA, USA) by applying a one-way analysis of variance (ANOVA) through post hoc Dunnett’s test for the multiple comparison. When Dunnett’s test was applied, the *p*-value was considered statistically significant to be less than 0.05. All data were expressed as the mean ± standard error.

## Figures and Tables

**Table 1:** Zonal heights of each group in GP of proximal tibia in rats.

	Control	rhGH 200 μg/kg (s.c.)	EEM 50 mg/kg (p.o.)	EEM 200 mg/kg (p.o.)
overall height of GP (μm)	350.3 ± 19.6	365.4 ± 17.3 ***	368.2 ± 27.7 *	368.0 ± 31.4 *
resting zone	22.0 ± 5.3	21.4 ± 5.2	22.3 ± 5.8	21.1 ± 4.9
proliferative zone	119.9 ± 11.5	127.3 ± 15.7 **	132.0± 12.2 **	125.8 ± 12.8
hypertrophic zone	198.5 ± 12.7	203.3 ± 17.7	207.5 ± 22.1	208.7 ± 19.1 *

Data are shown as mean ± SEM. The number of rats is eight per group; * *p* < 0.05, ** *p* < 0.01 and *** *p* < 0.001 vs. control.
